# Anaerobic antibiotic exposure and risk of maculopathy: a nationwide dual design study

**DOI:** 10.3389/fphar.2026.1840229

**Published:** 2026-08-21

**Authors:** Jiyeon Park, Seunghyun Cheon, Yu-Seon Choi, Jiyeong Kim, Seong Joon Ahn, Jee-Eun Chung

**Affiliations:** 1 College of Pharmacy and Institute of Pharmaceutical Science and Technology, Hanyang University-ERICA Campus, Ansan, Republic of Korea; 2 Department of Pre-Medicine, College of Medicine and Biostatistics Laboratory, Medical Research Collaborating Center, Hanyang University, Seoul, Republic of Korea; 3 Department of Ophthalmology, College of Medicine, Hanyang University Hospital, Hanyang University, Seoul, Republic of Korea

**Keywords:** anaerobes, antibiotics, claim data, dysbiosis, maculopathy

## Abstract

The pathogenesis of maculopathy remains incompletely understood, and emerging evidence implicates the gut–eye axis in retinal and macular diseases. Given that antibiotics with anti-anaerobic activity may disrupt gut microbial ecology, this study investigated their association with the risk of incident maculopathy. We conducted a nationwide population-based study using the National Health Insurance Service–National Sample Cohort of South Korea. A retrospective cohort study was used to assess cumulative systemic anaerobic antibiotic exposure during a 5-year baseline period and subsequent incident maculopathy from 2007 to 2019. After 1:1 propensity score matching, hazard ratios (HRs) and 95% confidence intervals (CIs) were estimated using Cox proportional hazards regression. A nested case-control study was also conducted using risk-set sampling and 1:10 propensity score matching. Anaerobic antibiotic exposure during the 365 days before the index date was assessed, and odds ratios (ORs) and 95% CIs were estimated using conditional logistic regression. Duration-response relationships were evaluated using formal trend analyses. Sensitivity analyses included age restriction, Fine-Gray competing risk models, extended exposure assessment windows, lag-time analyses, and a negative control comparator analysis using first-generation cephalosporins. In the retrospective cohort study, 326,436 anaerobic antibiotic users were matched to 326,436 non-users. Anaerobic antibiotic use was associated with an increased risk of incident maculopathy in the fully adjusted model (HR, 1.07; 95% CI, 1.03–1.12), with a significant duration-response trend and the highest risk observed for ≥57 days of cumulative use (HR, 1.24; 95% CI, 1.09–1.42). In the nested case-control study, 55,776 cases were matched to 557,760 controls. Anaerobic antibiotic use within 365 days before the index date was associated with increased odds of maculopathy (OR, 1.04; 95% CI, 1.02–1.07), also showing a significant duration-response trend. Sensitivity analyses and first-generation cephalosporin comparator analyses, generally supported the robustness of the findings. Systemic exposure to antibiotics with anti-anaerobic activity was associated with an increased risk of incident maculopathy, with a duration-response pattern across cumulative exposure categories. These findings suggest further investigation into gut microbiome disruption and the gut-eye axis as potential pathways involved in maculopathy.

## 1 Introduction

Maculopathy refers to a group of retinal disorders affecting the macula, the central region of the retina responsible for high-resolution color and detailed vision (Pantelidou et al., 2024; Rehman et al., 2018). Because the macula plays a central role in visual acuity, color discrimination, and detailed visual perception, structural damage in this region may lead to substantial and potentially irreversible vision loss (Koksaldi et al., 2025; Gagliardi et al., 2019). Identifying modifiable factors associated with maculopathy is therefore important for prevention and early risk assessment.

Maculopathy is multifactorial, and several clinical and lifestyle factors, including age, sex, smoking, hypertension, diabetes, and metabolic disorders, have been associated with macular diseases (Babaker et al., 2025). However, these factors may not fully explain the development of macular pathologies, suggesting the need to consider additional systemic pathways. Recently, the gut–eye axis has gained attention as a potential framework for understanding retinal diseases, including maculopathy. Alterations in gut microbial composition and function may affect ocular tissues through immune, metabolic, and inflammatory pathways (Kammoun et al., 2024). Several human and animal studies, mostly in the context of macular degeneration, have reported reduced abundance of obligate anaerobic genera, including Blautia, Oscillibacter, and *Bacteroides*, compared with controls (Zysset-Burri et al., 2020; Zhang et al., 2023). These findings suggest that microbiome-related mechanisms may be relevant to broader pathological processes affecting the macular region beyond a degenerative disease entity.

Antibiotic exposure is a major external factor capable of disrupting the gut microbiome. In particular, antibiotics with activity against anaerobes may alter gut microbial ecology because obligate anaerobic bacteria constitute a dominant component of the healthy gut microbiota and contribute to gut homeostasis (Lamaudiere and Morozov, 2018). Disruption of these anaerobic communities may influence microbial metabolite production, immune regulation, and inflammatory responses, which are pathways potentially relevant to retinal and macular pathology. However, whether exposure to antibiotics with anti-anaerobic activity is associated with the development of maculopathy remains unclear.

Therefore, this study aimed to evaluate the association between exposure to antibiotics with anti-anaerobic activity and the risk of incident maculopathy using the National Health Insurance Service claims database of South Korea, with a retrospective cohort design and a nested case-control design applied as complementary approaches.

## 2 Methods

### 2.1 Data source

This study used the National Health Insurance Service–National Sample Cohort (NHIS-NSC), a large, population-based, de-identified database established by the National Health Insurance Service of South Korea (Lee et al., 2017; Rim et al., 2018; Kim et al., 2024). The NHIS administers a mandatory, universal national health insurance program as a single insurer. The NHIS-NSC comprises a nationally representative sample of approximately one million individuals and provides longitudinal follow-up data from 2002 to 2019. It includes detailed information on sociodemographic characteristics, healthcare utilization, including inpatient and outpatient visits, procedures, and diagnoses, and prescriptions. The study protocol was reviewed by the Institutional Review Board of Hanyang University, and the requirement for informed consent was waived (IRB No. HYUIRB-202505-004).

### 2.2 Study design

Two complementary study designs—a retrospective cohort design and a nested case-control design—were used to evaluate the association between anaerobic antibiotic exposure and incident maculopathy. The retrospective cohort design assessed cumulative anaerobic antibiotic use during the baseline exposure assessment period, whereas the nested case-control design evaluated antibiotic exposure before each case’s index date to capture more recent exposure closer to outcome occurrence.

#### 2.2.1 Retrospective cohort study

Patients from the NHIS-NSC were followed from 1 January 2007, the index date, until the earliest occurrence of maculopathy diagnosis, death, or 31 December 2019. We excluded patients without continuous eligibility for follow-up during the 2002–2006 baseline period, as well as those with prior maculopathy, ocular procedures, hydroxychloroquine or pentosan polysulfate use, or retinal diseases involving macular complications before the index date (Supplementary Tables S1,S2) (Hsu et al., 2021; Le and Shakoor, 2021).

#### 2.2.2 Nested case-control study

**FIGURE 1:**
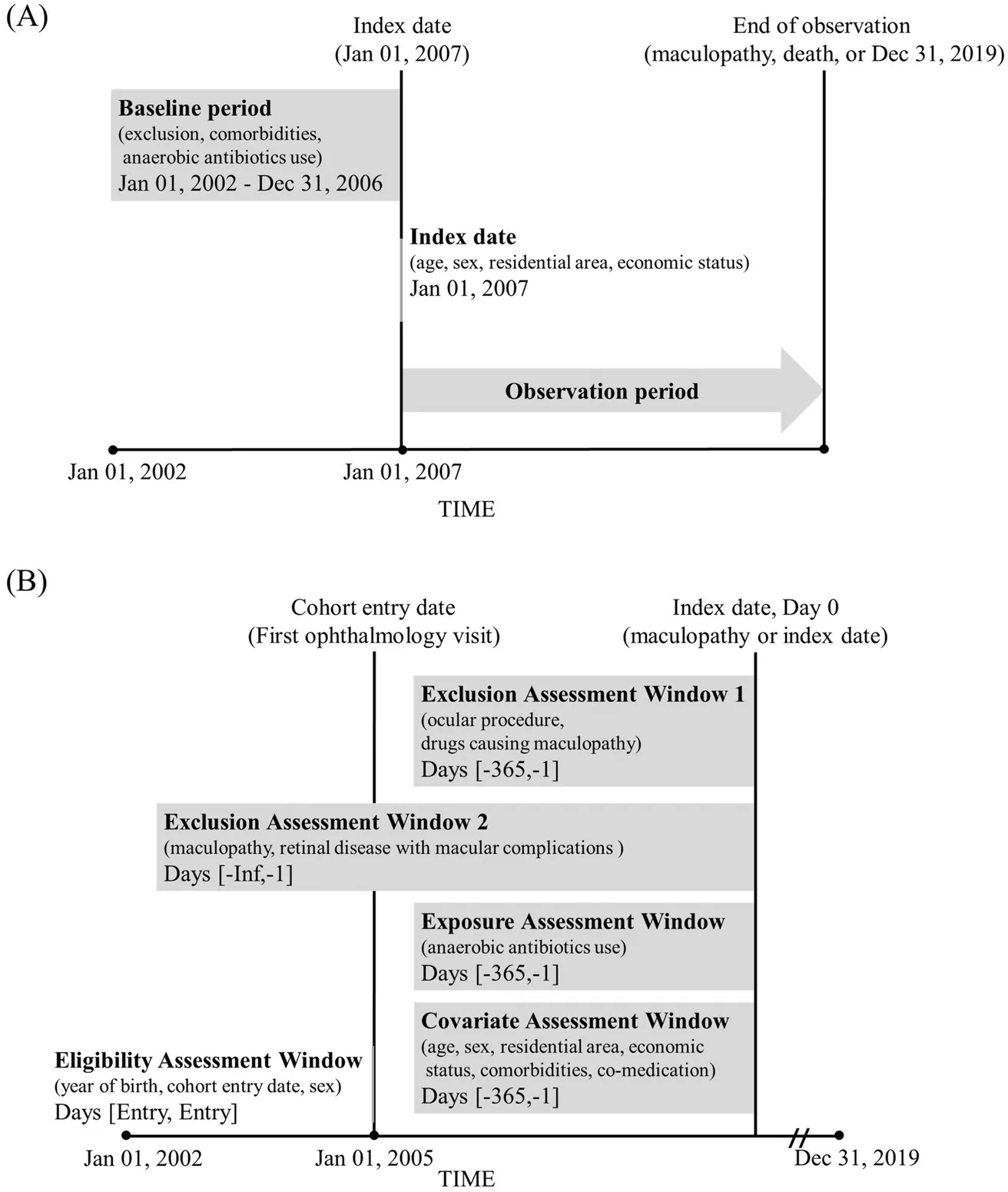
Study design for the retrospective cohort study **(A)** and the nested case-control study **(B)**.

The cohort comprised patients with ophthalmology visits between 2005 and 2019, who were followed from their first visit until incident maculopathy, death, or the end of the study period. Cases were defined as patients who developed incident maculopathy after cohort entry. Controls were selected using risk-set sampling at each case’s index date from patients who were still at risk and had not developed maculopathy by that date (Etminan et al., 2012). Controls were matched to each case on year of birth, sex, and cohort entry date (±90 days), with a maximum case-to-control ratio of 1:100. Subsequently, controls were excluded if they had a history of ocular procedures or use of maculopathy-inducing drugs within the prior 365 days, or if they had retinal diseases with macular complications before the index date. Finally, case-control sets were retained for analysis after propensity score matching at a 1:10 ratio. The overall study design is illustrated in Figure 1.

### 2.3 Outcome definition

In both study designs, the outcome was incident maculopathy, defined using ICD-10 codes H31.0 (chorioretinal scars), H35.3 (degeneration of macula and posterior pole), and H35.8 (other specified retinal disorders) adapted from Kwon et al. (2024). However, to enhance diagnostic accuracy and minimize potential misclassification, we applied a more stringent criterion requiring at least two claims with these codes. This approach allowed us to capture a broader disease spectrum involving the macular region rather than restricting the outcome to a single entity.

### 2.4 Exposure definition

We focused on systemic antibiotics with activity against anaerobes and identified 15 agents based on previous studies (Hollinger and Tobin, 2026). These included doripenem, ertapenem, imipenem, meropenem, panipenem, cefotetan, cefoxitin, ceftizoxime, flomoxef, clindamycin, metronidazole, amoxicillin/clavulanate, ampicillin/sulbactam, piperacillin/tazobactam, and moxifloxacin. For the retrospective cohort study, drug exposure was assessed during the baseline period from 1 January 2002, to 31 December 2006 (Lee et al., 2024). Patients with any use of anaerobic antibiotics during the baseline period were classified as users, and those without such use were classified as non-users. For each patient, cumulative exposure duration during the baseline period was calculated as the sum of prescription days for anaerobic antibiotics, regardless of whether prescriptions were continuous or intermittent. Users were then categorized into four exposure groups based on cumulative duration: 1–14 days, 15–28 days, 29–56 days, and ≥57 days.

For the nested case-control study, the 365-day period before the index date was defined as the exposure assessment window, during which use of anaerobic antibiotics was evaluated. Patients with any use of these agents during the exposure assessment window were classified as users, and those without such use were classified as non-users. Among users, cumulative exposure duration during the exposure assessment window was calculated using the same approach and categorized into three groups: 1–7 days, 8–28 days, and ≥29 days.

### 2.5 Covariates

Demographics and clinical characteristics, including age, sex, residential area, economic status, healthcare utilization—ophthalmology visits and hospitalization—comorbidities, and drug use, were assessed. Comorbidities were identified by the presence of ICD-10 diagnosis codes corresponding to the following diseases: diabetes mellitus, cataract, glaucoma, hypertension, dyslipidemia, and chronic kidney disease. Drug exposure included the use of oral or ophthalmic corticosteroids (Supplementary Table S3). In the retrospective cohort study, demographic characteristics were assessed at the index date, while healthcare utilization, comorbidities and corticosteroid use were assessed during the baseline period. In the nested case-control study, age, sex, residential area, and economic status were evaluated at the index date. Healthcare utilization, comorbidities and drug use were examined within the covariate assessment window, which was defined as the 365 days before the index date.

### 2.6 Statistical analysis

Baseline characteristics were presented as means with standard deviations for continuous variables and as frequencies with percentages for categorical variables. Differences between two groups were assessed using standardized mean differences (SMDs). Propensity score matching was performed based on age, sex, residential area, economic status, healthcare utilization, comorbidities, and use of oral or ophthalmic corticosteroid. Matching was performed without replacement using a caliper width of 0.15, and subjects who could not be matched within the caliper were excluded from the matched analytic population. Matching quality was considered adequate when all matching variables demonstrated SMDs of 0.2 or less (Andrade, 2020).

In the retrospective cohort study, non-users and users were matched 1:1 using propensity scores to construct two groups with similar characteristics. Cox proportional hazards regression was used as the primary analysis to estimate hazard ratios (HRs) and 95% confidence intervals (CIs) for incident maculopathy in the propensity score-matched cohort. The proportional hazards assumption was assessed using Schoenfeld residuals, and no significant violation was observed in the global test or variable-specific tests. The primary comparison was performed between anaerobic antibiotic users and non-users. Additional analyses stratified users by cumulative exposure duration to evaluate the duration-response relationship. Three Cox models were constructed: Model 1 was the crude model without covariate adjustment, Model 2 was adjusted for the variables used in propensity score matching, and Model 3 was further adjusted for major infectious diagnoses, including respiratory tract infections, intra-abdominal infections, urinary tract infections, intestinal infections, and skin, soft tissue, bone, and joint infections (Lee et al., 2024). To formally assess the duration-response relationship, cumulative exposure duration was modeled as an ordinal variable, and a formal trend analysis was performed.

Moreover, Kaplan-Meier cumulative incidence curves were plotted to visualize unadjusted time-to-event outcomes, and differences between groups were evaluated using the two-sided log-rank test. In addition, adjusted cumulative incidence curves were generated from the Cox proportional hazards models to visualize covariate-adjusted differences in the cumulative incidence of maculopathy between groups (Denz et al., 2023).

In the nested case-control study, propensity score matching was performed to match cases to controls at a 1:10 ratio. Conditional logistic regression analysis was conducted to account for the matched design and to estimate odds ratios (ORs) and 95% CIs for the association between anaerobic antibiotics use and maculopathy onset. Three conditional logistic regression models were constructed using the same adjustment scheme as in the cohort analysis.

All statistical analyses were performed using SAS Enterprise Guide, version 8.3 (SAS Institute Inc., Cary, NC, United States) and R software, version 4.3.0 (R Foundation for Statistical Computing, Vienna, Austria).

### 2.7 Sensitivity and additional analysis

We conducted sensitivity analyses to assess the robustness of the primary findings. In both the retrospective cohort and nested case-control studies, we restricted the study population to individuals aged ≥40 years at the index date, given that the prevalence of maculopathy increases substantially from age 40 (Ma et al., 2024).

For the retrospective cohort study, we additionally performed a competing risk analysis using the Fine-Gray subdistribution hazard model to account for death as a competing event during the long follow-up period. For the nested case-control study, we varied the exposure assessment window from 1 year to 2 and 3 years before the index date. In addition, lag-time analyses were performed by excluding antibiotic exposures occurring within 30, 90, and 180 days before the index date to reduce potential reverse causation.

Additionally, a comparator analysis using first-generation cephalosporins as a negative control was performed. The first-generation cephalosporins included cefadroxil, cefatrizine, cefazolin, cefradine, cefroxadine, ceftezole, cephacetrile, cephalexin, cephalothin, cephapirin, and cephazedone.

## 3 Results

### 3.1 Study participants and characteristics

#### 3.1.1 Retrospective cohort study

**FIGURE 2:**
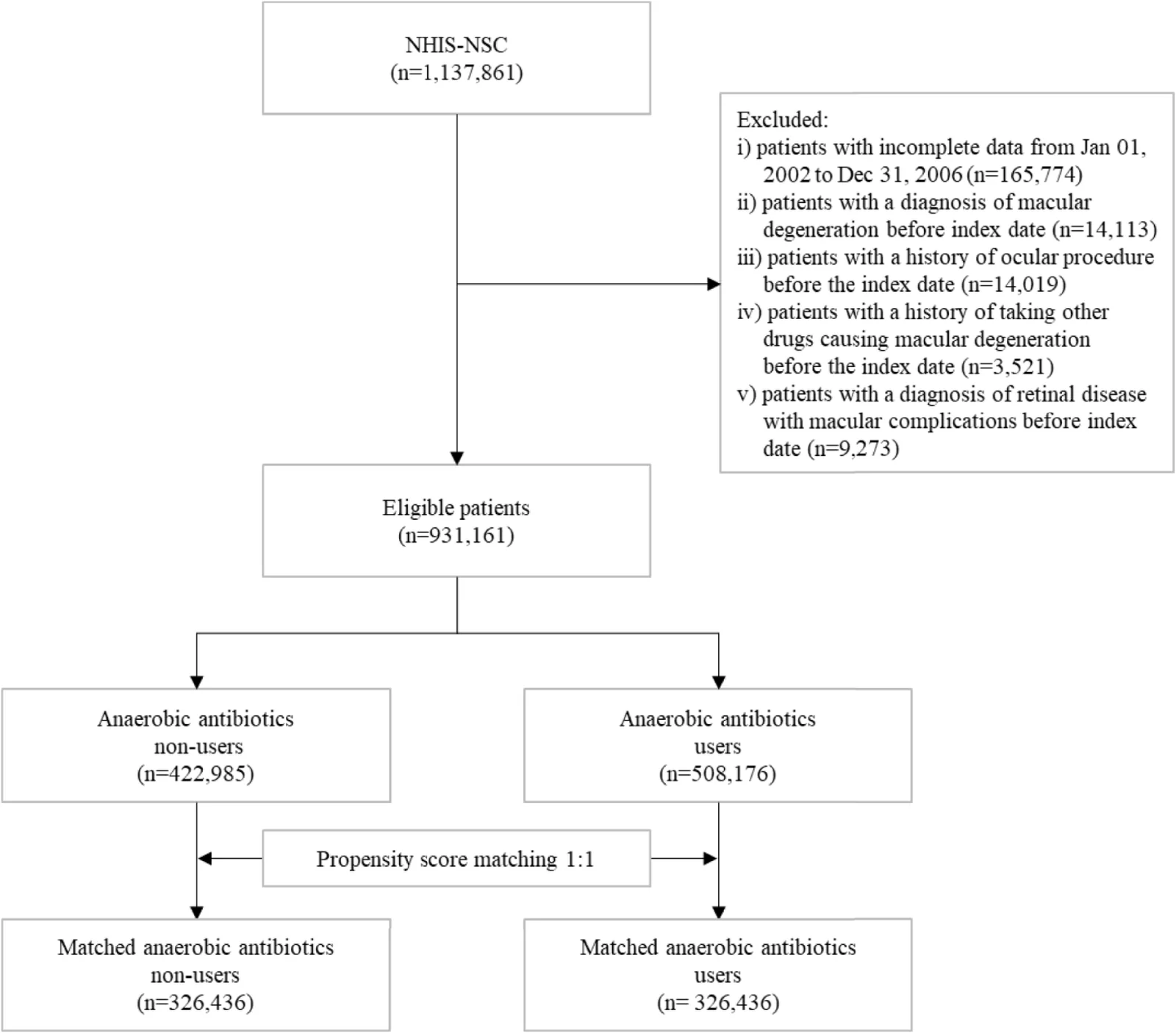
Flowchart for screening eligible patients in the retrospective cohort.

Among 1,137,861 individuals in the initial NHIS-NSC population, 165,774 who could not be continuously followed for the full 5-year period from 1 January 2002, to 31 December 2006, were excluded. An additional 14,113 patients with a diagnosis of maculopathy before the index date were excluded. Subsequently, 14,019 patients with a history of ocular procedures and 3,521 patients who had used hydroxychloroquine or pentosan polysulfate before the index date were excluded. Finally, 9,273 patients with retinal diseases associated with macular complications were excluded. The screening process for identifying eligible patients is presented in Figure 2.

As a result, the analytic cohort included 422,985 anaerobic antibiotic users and 508,176 non-users, totaling 931,161 patients before propensity score matching. Before matching, several baseline characteristics differed between anaerobic antibiotic users and non-users, particularly age, corticosteroid use, and healthcare utilization. After matching, the analytic cohort included 326,436 anaerobic antibiotic users and an equal number of non-users. Baseline characteristics were generally well balanced after matching, with standardized mean differences below 0.1 for most covariates (Table 1). A residual imbalance was observed for prior hospitalization, with SMDs ranging from 0.100 to 0.167.

**TABLE 1:** Baseline characteristics for anaerobic antibiotic users and non-users in the retrospective cohort study.

Characteristics	Before matching			After matching		
	Non-user (n = 422,985)	User (n = 508,176)	SMD	Non-user (n = 326,436)	User (n = 326,436)	SMD
	n (%)	n (%)		n (%)	n (%)	
Mean age (SD)	41.1 (17.4)	34.0 (18.9)	0.389	39.3 (17.3)	40.2 (18.0)	0.052
Sex			0.241	​		<0.001
Male	240,743 (56.9)	228,531 (45.0)		160,164 (49.1)	160,273 (49.1)	
Female	182,242 (43.1)	279,645 (55.0)		172,911 (53.0)	172,802 (52.9)	
Residential area						
Metropolitan areas	202,301 (47.8)	233,725 (46.0)	0.037	153,851 (47.1)	155,030 (47.5)	0.007
Urban areas	180,693 (42.7)	233,700 (46.0)	0.066	142,906 (43.8)	137,927 (42.3)	0.031
Rural areas	39,991 (9.5)	40,751 (8.0)	0.051	29,679 (9.1)	33,479 (10.3)	0.039
Economic status						
High	93,670 (22.1)	97,841 (19.3)	0.066	68,045 (20.8)	75,365 (23.1)	0.054
Middle	160,454 (37.9)	187,835 (37.0)	0.072	122,465 (37.5)	126,691 (38.8)	0.027
Low	147,107 (34.8)	206,221 (40.6)	0.020	122,585 (37.6)	109,577 (33.6)	0.083
Unknown	21,754 (5.1)	16,279 (3.2)	0.120	13,341 (4.1)	14,803 (4.5)	0.022
Comorbidity						
Diabetes mellitus	39,946 (9.4)	52,846 (10.4)	0.032	34,628 (10.6)	43,319 (13.3)	0.082
Glaucoma	28,037 (6.6)	45,257 (8.9)	0.085	26,123 (8.0)	32,171 (9.9)	0.065
Cataract	12,252 (2.9)	14,609 (2.9)	0.001	10,676 (3.3)	12,754 (3.9)	0.034
Hypertension	61,741 (14.6)	68,370 (13.5)	0.033	50,665 (15.5)	58,481 (17.9)	0.064
Dyslipidemia	49,275 (11.6)	69,128 (13.6)	0.059	43,921 (13.5)	55,327 (16.9)	0.097
Chronic kidneydisease	1,141 (0.3)	1,522 (0.3)	0.006	959 (0.3)	1,311 (0.4)	0.018
Ophthalmology visit						
0	250,649 (59.3)	197,397 (38.8)	0.409	164,219 (50.3)	154,777 (47.4)	0.058
1–2	115,253 (27.2)	175,467 (34.5)	0.157	106,429 (32.6)	104,018 (31.9)	0.016
≥3	57,083 (13.5)	135,312 (26.6)	0.324	55,788 (17.1)	67,641 (20.7)	0.093
Hospitalization						
0	353,950 (83.7)	360,889 (71.0)	0.300	260,270 (79.7)	236,997 (72.6)	0.167
1	48,772 (11.5)	95,820 (18.9)	0.202	46,418 (14.2)	58,345 (17.9)	0.100
≥2	20,263 (4.8)	51,467 (10.1)	0.200	19,748 (6.0)	31,094 (9.5)	0.130
Drug use						
Corticosteroid	222,187 (52.5)	404,357 (79.6)	0.596	281,009 (66.8)	225,785 (69.2)	0.051

SD, standard deviation; SMD, standardized mean difference.

#### 3.1.2 Nested case-control study

**FIGURE 3:**
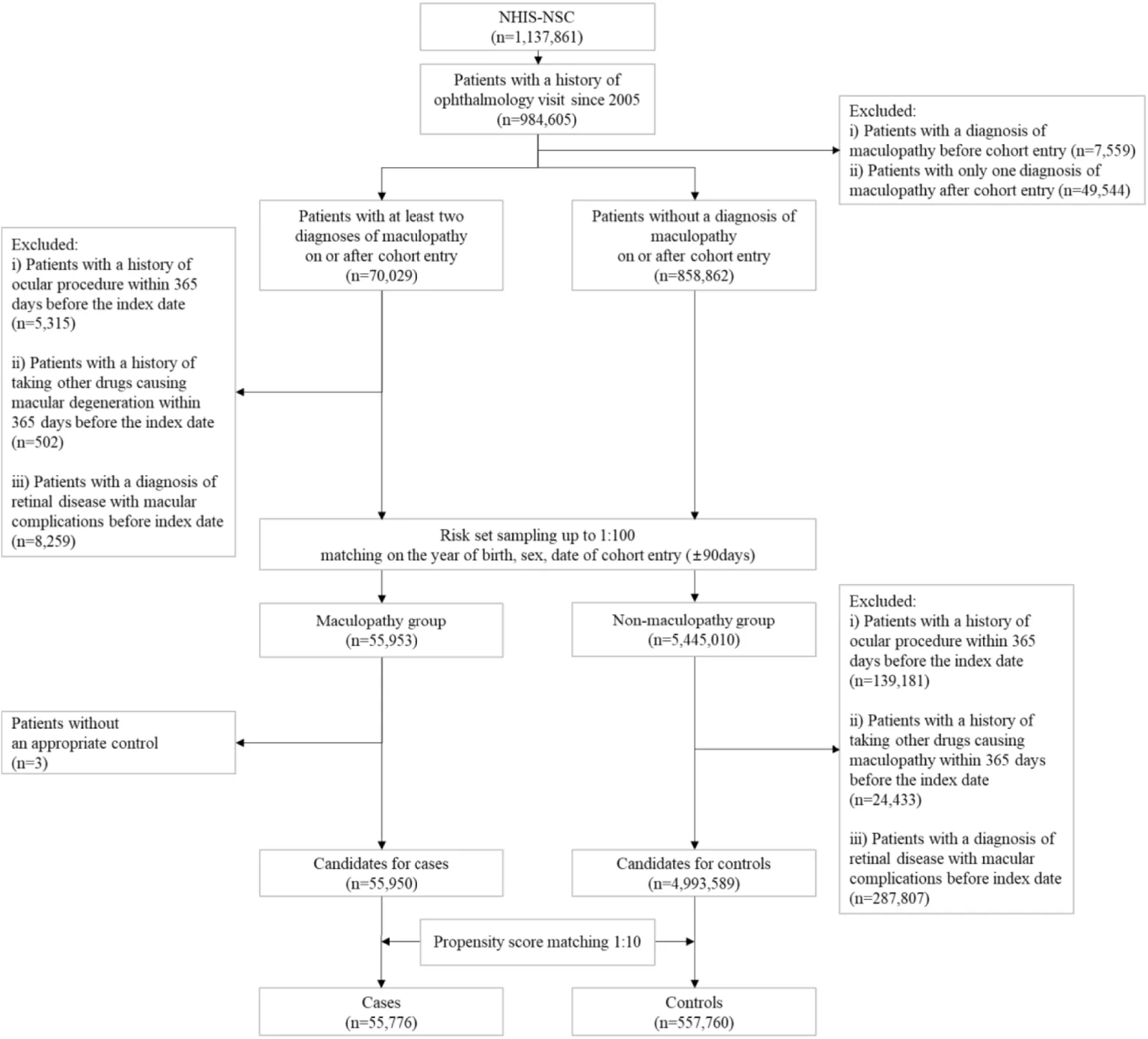
Flowchart for screening eligible patients in the nested case-control study.

Of the 1,137,861 patients included in the NHIS-NSC, approximately 86.5% had at least one ophthalmology visit between 1 January 2005 and 31 December 2019. After excluding 7,559 individuals with prior maculopathy and 49,544 individuals with only a single diagnosis of maculopathy, 70,029 incident cases and 858,862 non-cases remained. Among the incident cases, sequential exclusions were then applied for 5,315 individuals with ocular procedures, 502 using maculopathy-inducing drugs, and 8,259 with retinal diseases, yielding 55,953 final cases. Risk-set sampling identified 5,445,010 potential controls, of whom 4,993,589 remained eligible after applying the same exclusion criteria. After excluding 3 cases without sufficient controls, propensity score matching achieved a 1:10 ratio (55,776 cases; 557,760 controls). The selection process is shown in Figure 3.

Table 2 summarizes the baseline characteristics of the cases and controls before and after propensity score matching. Before matching, imbalances were observed for several variables, including glaucoma, cataract, and ophthalmology visits. After matching, all variables used for matching were well balanced between the two groups, with all SMDs below 0.05.

**TABLE 2:** Characteristics for the populations included in the nested case-control study.

Characteristics	Before matching			After matching		
	Cases (n = 55,776)	Controls (n = 4,993,589)	SMD	Cases (n = 55,776)	Controls (n = 557,760)	SMD
	n (%)	n (%)		n (%)	n (%)	
Mean age (SD)	56.7 (18.9)	55.1 (19.0)	0.084	56.7 (18.9)	57.1 (18.6)	0.022
Sex			<0.001	​		0.007
Male	23,717 (42.5)	2,125,671 (42.6)		23,717 (42.5)	235,203 (42.2)	
Female	32,059 (57.5)	2,867,918 (57.4)		32,059 (57.5)	322,557 (57.8)	
Residential area						
Metropolitan areas	26,527 (47.6)	2,232,622 (44.7)	0.057	26,527 (47.6)	268,532 (48.1)	0.012
Urban areas	24,160 (43.3)	2,221,735 (44.5)	0.024	24,160 (43.3)	241,642 (43.3)	<0.001
Rural areas	4,857 (8.7)	504,117 (10.1)	0.046	4,857 (8.7)	45,846 (8.2)	0.018
Unknown	232 (0.4)	35,115 (0.7)	0.034	232 (0.4)	1,740 (0.3)	0.018
Economic status						
High	12,068 (21.6)	1,097,516 (22.0)	0.008	12,068 (21.6)	118,428 (21.2)	0.010
Middle	17,093 (30.6)	1,634,200 (32.7)	0.044	17,093 (30.6)	170,024 (30.5)	0.004
Low	24,021 (43.1)	2,104,107 (42.1)	0.019	24,021 (43.1)	243,722 (43.7)	0.013
Unknown	2,594 (4.7)	157,766 (3.2)	0.085	2,594 (4.7)	25,586 (4.6)	0.003
Comorbidity						
Diabetes mellitus	10,862 (19.5)	733,989 (14.7)	0.127	10,862 (19.5)	108,541 (19.5)	<0.001
Glaucoma	10,525 (18.9)	515,526 (10.3)	0.244	10,525 (18.9)	104,922 (18.8)	0.002
Cataract	10,001 (17.9)	466,516 (9.3)	0.252	10,001 (17.9)	100,074 (17.9)	<0.001
Hypertension	20,794 (37.3)	1,623,929 (32.5)	0.100	20,794 (37.3)	210,675 (37.8)	0.010
Dyslipidemia	19,034 (34.1)	1,393,220 (27.9)	0.135	19,034 (34.1)	190,869 (34.2)	0.002
Chronic kidneydisease	560 (1.0)	32,791 (0.7)	0.038	560 (1.0)	4,293 (0.8)	0.026
Ophthalmology visit						
0	23,958 (43.0)	2,496,908 (50.0)	0.141	23,958 (43.0)	240,217 (43.1)	0.002
1–2	17,952 (32.2)	1,836,565 (36.8)	0.095	17,952 (32.2)	176,760 (31.7)	0.011
≥3	13,866 (24.9)	660,116 (13.2)	0.342	13,866 (24.9)	140,783 (25.2)	0.009
Hospitalization						
0	46,267 (83.0)	4,276,406 (85.6)	0.077	46,267 (83.0)	470,102 (84.3)	0.036
1	6,039 (10.8)	463,013 (9.3)	0.054	6,039 (10.8)	57,604 (10.3)	0.016
≥2	3,470 (6.2)	254,170 (5.1)	0.051	3,470 (6.2)	30,054 (5.4)	0.037
Drug use						
Corticosteroid	15,984 (28.7)	1,172,802 (23.5)	0.118	15,984 (28.7)	158,012 (28.3)	0.007

SD, standard deviation; SMD, standardized mean difference.

### 3.2 Primary analysis: associations from cohort and nested case-control studies

#### 3.2.1 Retrospective cohort study

A total of 41,115 maculopathy events occurred among anaerobic antibiotic users and 34,674 among non-users. The incidence rates were 1.03 and 0.86 per 100 person-years, respectively, corresponding to an absolute incidence rate difference of 0.17 per 100 person-years. In the stratified Cox proportional hazards regression analysis, anaerobic antibiotic use was significantly associated with an increased risk of maculopathy compared with non-use, with HRs of 1.20 (95% CI, 1.18–1.22) in Model 1, 1.14 (95% CI, 1.10–1.19) in Model 2, and 1.07 (95% CI, 1.03–1.12) in Model 3 (Table 3).

**TABLE 3:** Maculopathy risk according to anaerobic antibiotic use in the matched cohort.

Group	Group size (n)	Events (n)	Follow-up time (person-years)	HR (Model 1)^a^ (95% CI)	HR (Model 2)^b^ (95% CI)	HR (Model 3)^c^ (95% CI)	ARDs^d^ (%)	
							At 5 years	At 10 years
Non-users	326,436	34,674	4,010,572.3	Ref	Ref	Ref	Ref	Ref
Users	326,436	41,115	3,981,421.2	1.20 (1.18–1.22)	1.14 (1.10–1.19)	1.07 (1.03–1.12)	0.13	0.35
1–14 days	245,020	30,923	2,990,143.3	1.19 (1.17–1.21)	1.13 (1.09–1.18)	1.07 (1.02–1.11)	0.11	0.30
15–28 days	51,206	6,790	621,191.7	1.27 (1.23–1.32)	1.20 (1.13–1.27)	1.11 (1.04–1.18)	0.16	0.42
29–56 days	21,501	2,681	261,913.8	1.26 (1.18–1.33)	1.34 (1.23–1.45)	1.22 (1.12–1.33)	0.30	0.80
≥57 days	8,709	721	108,172.4	0.94 (0.85–1.05)	1.39 (1.21–1.58)	1.24 (1.09–1.42)	0.33	0.88
Trend analysis (per category increase)				1.11 (1.10–1.13)	1.09 (1.07–1.12)	1.06 (1.04–1.08)	—	—

ARD, absolute risk difference; CI, confidence interval; HR, hazard ratio.

^a^ Model 1 was the crude model without covariate adjustment.

^b^ Model 2 was adjusted for age, sex, residential area, economic status, ophthalmology visits, hospitalization, diabetes mellitus, glaucoma, cataract, hypertension, dyslipidemia, chronic kidney disease, and use of oral or ophthalmic corticosteroids.

^c^ Model 3 was adjusted for all variables included in Model 1 plus major infectious diagnoses, including respiratory tract infections, intra-abdominal infections, urinary tract infections, intestinal infections, and skin, soft tissue, bone, and joint infections.

^d^ Absolute risk differences at 5 and 10 years were derived from the adjusted cumulative incidence curves estimated using Cox model 3.

When anaerobic antibiotic users were categorized according to cumulative exposure duration, the risk of maculopathy was higher across all duration categories than among non-users in Model 3, the fully adjusted model (Table 3). Compared with non-users, the HRs were 1.07 (95% CI, 1.02–1.11) for 1–14 days, 1.11 (95% CI, 1.04–1.18) for 15–28 days, 1.22 (95% CI, 1.12–1.33) for 29–56 days, and 1.24 (95% CI, 1.09–1.42) for ≥57 days of use. In the trend analysis for Model 3, cumulative exposure duration showed a significant positive trend with incident maculopathy, with an HR of 1.06 (95% CI, 1.04–1.08) per increase in duration category.

**FIGURE 4:**
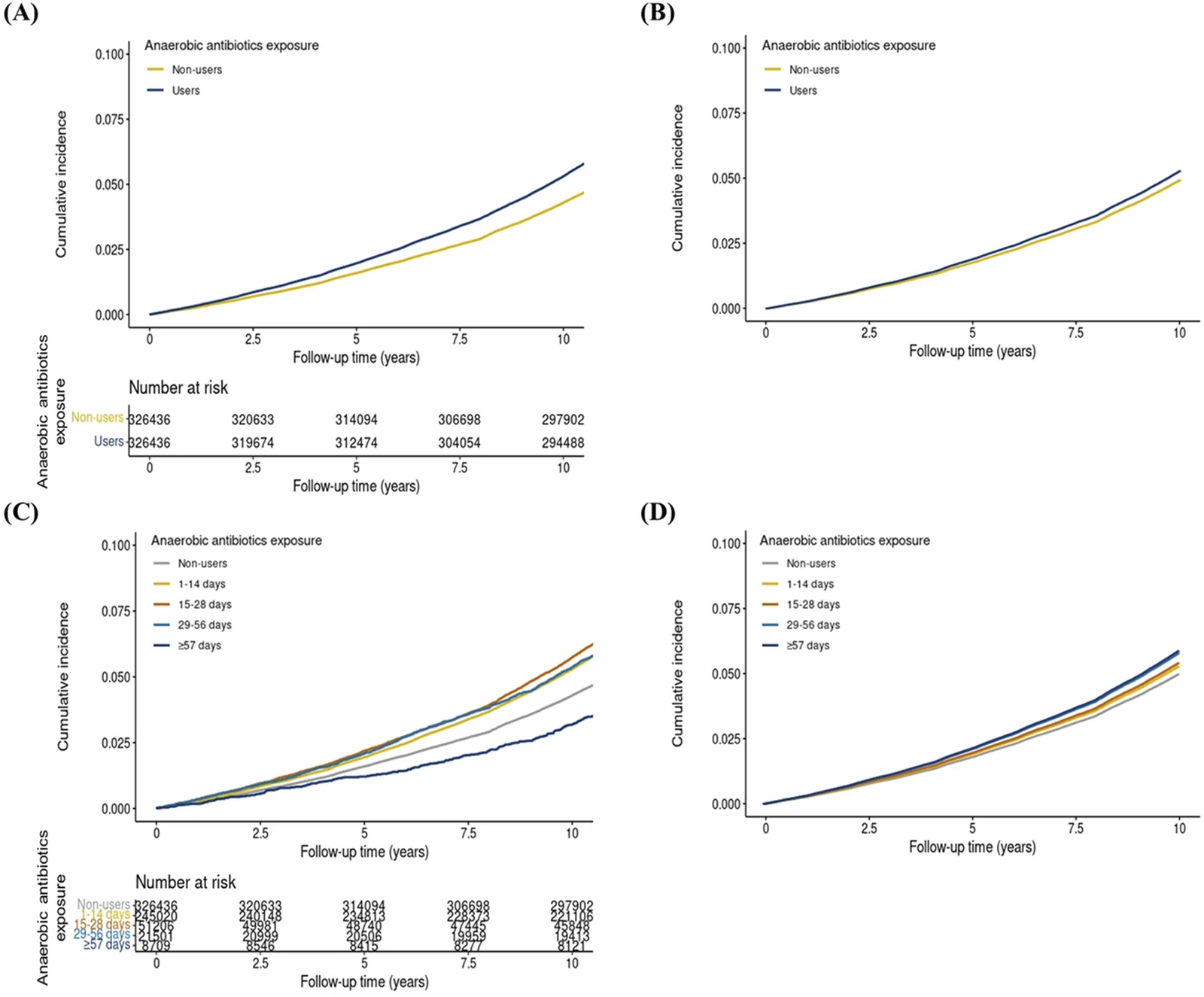
Cumulative incidence curves for incident maculopathy **(A)** crude user vs. non-user comparison; **(B)** adjusted user vs. non-user comparison; **(C)** crude duration-stratified comparison; and **(D)** adjusted duration-stratified comparison.

The crude Kaplan-Meier cumulative incidence curves showed a higher cumulative incidence of maculopathy among anaerobic antibiotic users than among non-users (log-rank p < 0.0001; Figure 4A). This pattern was consistent in the adjusted cumulative incidence curves derived from the Cox proportional hazards model, in which users continued to show a higher cumulative incidence than non-users (Figure 4B). The absolute risk differences between users and non-users were 0.38 percentage points at 5 years and 1.01 percentage points at 10 years in the crude curves, and 0.13 percentage points at 5 years and 0.35 percentage points at 10 years in the adjusted curves.

When users were stratified according to cumulative exposure duration, the crude cumulative incidence curves showed a stepwise increase in maculopathy risk for the 1–14, 15–28, and 29–56 days groups compared with non-users, whereas the ≥57 days group showed a lower cumulative incidence than non-users (Figure 4C). In contrast, the adjusted cumulative incidence curves showed a more consistent duration-response pattern, with the ≥57 days group demonstrating a higher cumulative incidence than non-users after adjustment for covariates (Figure 4D). The adjusted absolute risk differences compared with non-users 0.11 percentage points for 1–14 days, 0.16 for 15–28 days, 0.30 for 29–56 days, and 0.33 for ≥57 days at 5 years, and 0.30, 0.42, 0.80, and 0.88 percentage points, respectively, at 10 years (Table 3).

#### 3.2.2 Nested case-control study

Approximately 30.1% of cases and 28.5% of controls had used anaerobic antibiotics within 1 year before the index date. Conditional logistic regression showed a significant association between anaerobic antibiotic use and maculopathy (Table 4). The crude OR in Model 1 was 1.13 (95% CI, 1.11–1.16). The adjusted OR was 1.11 (95% CI, 1.08–1.13) in Model 2, which adjusted for the matching variables, and 1.04 (95% CI, 1.02–1.07) in Model 3, which further adjusted for major infectious diagnoses.

**TABLE 4:** Maculopathy risk according to anaerobic antibiotic use in the nested case-control study.

Group	Control (n)	Case (n)	OR (Model 1)^a^ (95% CI)	OR (Model 2)^b^ (95% CI)	OR (Model 3)^c^ (95% CI)
Non-users	398,686	38,973	Ref	Ref	Ref
Users	159,074	16,803	1.13 (1.11–1.16)	1.11 (1.08–1.13)	1.04 (1.02–1.07)
1–7 days	84,807	8,871	1.10 (1.07–1.13)	1.08 (1.05–1.11)	1.03 (1.00–1.06)
8–28 days	64,187	6,905	1.18 (1.14–1.21)	1.13 (1.10–1.17)	1.07 (1.03–1.10)
≥29 days	10,080	1,027	1.24 (1.15–1.34)	1.15 (1.06–1.25)	1.08 (1.01–1.16)
Trend analysis (per category increase)			1.08 (1.07–1.10)	1.06 (1.05–1.08)	1.03 (1.02–1.04)

CI, confidence interval; OR, odds ratio.

^a^ Model 1 was the crude model without covariate adjustment.

^b^ Model 2 was adjusted for age, sex, residential area, economic status, ophthalmology visits, hospitalization, diabetes mellitus, glaucoma, cataract, hypertension, dyslipidemia, chronic kidney disease, and use of oral or ophthalmic corticosteroids.

^c^ Model 3 was adjusted for all variables included in Model 1 plus major infectious diagnoses, including respiratory tract infections, intra-abdominal infections, urinary tract infections, intestinal infections, and skin, soft tissue, bone, and joint infections.

When categorized by cumulative exposure duration, the odds of maculopathy were generally higher with longer antibiotic exposure (Table 4). After full adjustment in Model 3, compared with non-use, the adjusted ORs were 1.03 (95% CI, 1.00–1.06), 1.07 (95% CI, 1.03–1.10), and 1.08 (95% CI, 1.01–1.16) for 1–7, 8–28, and ≥29 days of use, respectively. A significant positive trend was observed across increasing duration categories in Model 3 (OR per category increase, 1.03; 95% CI, 1.01–1.04).

### 3.3 Sensitivity and additional analysis

#### 3.3.1 Retrospective cohort study

In the matched cohort of individuals aged ≥40 years, the number of users and non-users decreased to 156,921 in each group. The association between anaerobic antibiotic use and maculopathy remained generally consistent with the primary analysis (Supplementary Table S5). In Model 3, the fully adjusted model, anaerobic antibiotic use was associated with an increased risk of maculopathy compared with non-use (HR, 1.09; 95% CI, 1.04–1.15). A similar duration-response pattern was observed, with a significant positive trend across increasing duration categories (HR per category increase, 1.05; 95% CI, 1.02–1.08). In the Fine-Gray competing risk model, the estimates were generally attenuated compared with those from the Cox proportional hazards model; however, cumulative anaerobic antibiotic exposure remained significantly associated with a higher risk of incident maculopathy (Supplementary Table S6).

In the negative control comparator analysis, first-generation cephalosporin exposure was not significantly associated with incident maculopathy compared with non-users after full adjustment for matching variables and major infectious diagnoses (HR, 1.00; 95% CI, 0.96–1.05). In the duration-stratified analysis, no clear duration-response pattern was observed, with fully adjusted HRs of 1.00 (95% CI, 0.95–1.04), 1.06 (95% CI, 0.98–1.13), 1.09 (95% CI, 0.99–1.19), and 1.04 (95% CI, 0.87–1.25) for 1–14, 15–28, 29–56, and ≥57 days of use, respectively.

#### 3.3.2 Nested case-control study

In the nested case-control analysis restricted to individuals aged ≥40 years, the association between anaerobic antibiotic use and maculopathy remained generally consistent with the primary nested case-control analysis (Supplementary Table S7). In the fully adjusted model, anaerobic antibiotic use was associated with increased odds of maculopathy compared with non-use (OR, 1.04; 95% CI, 1.02–1.06). A similar duration-response pattern was observed, with a significant positive trend across increasing duration categories (OR per category increase, 1.02; 95% CI, 1.01–1.04).

In sensitivity analyses extending the exposure assessment window from 1 year to 2 and 3 years, the association between anaerobic antibiotic use and maculopathy was generally consistent with the primary analysis (Supplementary Table S8). In the lag-time sensitivity analyses, some duration-specific estimates were attenuated and did not reach statistical significance after applying lag times of 30, 90, and 180 days. Nevertheless, the formal trend analyses remained significant across all lag-time definitions in the fully adjusted model, with an OR of 1.03 per increase in exposure-duration category (Supplementary Table S9).

In line with the cohort findings, the nested case-control analysis showed no significant association between first-generation cephalosporin exposure and maculopathy in the fully adjusted model (OR, 0.99; 95% CI, 0.96–1.02). The corresponding ORs were 0.97 (95% CI, 0.94–1.01), 1.01 (95% CI, 0.96–1.05), and 1.09 (95% CI, 0.94–1.27) for 1–7, 8–28, and ≥29 days of use, respectively.

## 4 Discussion

Through a complementary nested case-control study and retrospective cohort study design, we consistently demonstrated that individuals exposed to anaerobic antibiotics carry a significantly elevated risk of maculopathy compare to unexposed individuals. The risk of maculopathy increased progressively with cumulative days of anaerobic antibiotics use.

Several established factors, including cataract, diabetes, and hypertension, have been reported to be associated with maculopathy (Babaker et al., 2025; Chakravarthy et al., 2010; Heesterbeek et al., 2020). Therefore, these comorbidities were taken into account, and matching was performed to balance baseline characteristics between anaerobic antibiotic users and non-users and to better evaluate the independent association between antibiotic exposure and maculopathy. Beyond these established clinical factors, the gut microbiome has recently emerged as a potential contributor to retinal and macular diseases.

In the field of ophthalmology, several studies have investigated the relationship between antibiotic-associated dysbiosis and ophthalmic conditions, such as keratoconjunctivitis and myopia, suggesting the potential relevance of the gut-eye axis in ocular disease (Ikeda et al., 2025; Wang et al., 2019; Zhang and Mo, 2023). In a healthy gut, obligate anaerobes maintain intestinal homeostasis, whereas anaerobic antibiotics can selectively deplete these dominant taxa and enable the proliferation of facultative anaerobes and opportunistic pathogens (Lamaudiere and Morozov, 2018; Kriss et al., 2018). This microbial shift may theoretically lead to the loss of beneficial metabolites, such as short-chain fatty acids (SCFAs), and compromise intestinal epithelial barrier integrity (Kammoun et al., 2024). In experimental and laboratory models, increased intestinal permeability may allow the translocation of bacterial endotoxins, including lipopolysaccharide (LPS), into the systemic circulation, thereby triggering systemic inflammatory and immune responses (Kammoun et al., 2024; Zhang and Mo, 2023; Zheng et al., 2025). These circulating microbial products and inflammatory mediators may, in turn, be relevant to macular pathology by contributing to blood-retinal barrier (BRB) dysfunction, a key feature implicated in various retinal diseases (Zhang and Mo, 2023).

For instance, experimental data suggest that systemic LPS can activate retinal microglia and impair retinal pigment epithelial (RPE) cells, thereby promoting retinal inflammation, oxidative stress, and cellular dysfunction (Leung et al., 2009; Xie and Lin, 2025). Conversely, SCFAs have been shown in animal models to attenuate intraocular inflammation, consistent with clinical observations of reduced SCFA-producing anaerobes in patients with ophthalmic diseases (Chen et al., 2021; Ciurariu et al., 2025). Collectively, while these biological pathways remain speculative and require further experimental validation in human cohorts, they provide a plausible mechanistic framework that aligns with our epidemiological findings.

Moir et al. investigated the association between antibiotic use and macular degeneration using U.S. Medicare claims data (Moir et al., 2023). They classified antibiotics according to chemical structure, such as penicillins, cephalosporins, and fluoroquinolones, and reported that aminoglycosides and fluoroquinolones were associated with the highest risk of macular degeneration (Moir et al., 2023). However, their study included patients with a history of ocular procedures or intravitreal injections, and chemical structure-based classification may not fully reflect differences in antimicrobial spectrum. In addition, the outcome was limited to macular degeneration, whereas microbiome-mediated mechanisms may plausibly affect a broader spectrum of macular pathologies. Given these issues, we excluded patients with a history of ocular procedures and focused on anaerobic antibiotics to refine the spectrum-based comparison. We also evaluated broad maculopathy rather than macular degeneration alone, aiming to capture overall macular diseases involving the macular region. This approach allowed us to assess the association across macular diseases more broadly; however, because this broader definition may introduce some degree of outcome heterogeneity, the findings should be interpreted with caution within the scope of this outcome definition. Considering that the gut microbiome varies by ethnicity and dietary habits, this association should be evaluated in diverse populations (Brooks et al., 2018). Therefore, the present study examined the association between anaerobic antibiotic use and maculopathy in a Korean population.

Our study further evaluated the association between anaerobic antibiotic use and maculopathy using both a retrospective cohort design and a nested case-control analysis. The retrospective cohort design allowed us to incorporate the temporal dimension of follow-up and to estimate HRs according to cumulative exposure duration (Sessler and Imrey, 2015). In this analysis, anaerobic antibiotic exposure was assessed over a 5-year baseline period to examine whether long-term or repeated exposure was associated with subsequent maculopathy. After adjustment for potential confounders, including demographic factors, comorbidities, healthcare utilization, concomitant medications, and major infectious diagnoses, the HRs showed an increasing pattern with longer cumulative exposure duration. In particular, among patients with 57 days or more of cumulative anaerobic antibiotic use, the HR changed from 0.94 in the crude model to 1.24 in the fully adjusted model. These findings suggest that, after accounting for measured confounding and clinical indications related to antibiotic use, longer cumulative exposure was associated with a higher risk of maculopathy. Major infectious diagnoses were included as adjustment variables rather than matching variables because they are often transient clinical conditions related to the indication for antibiotic prescription, whereas the variables used for matching represented more stable baseline characteristics. This approach allowed us to balance underlying patient characteristics through matching while additionally accounting for infectious indications in the outcome model. Furthermore, formal trend analysis demonstrated a gradual increase in risk with longer cumulative exposure duration, further supporting a duration-response relationship between anaerobic antibiotic use and maculopathy.

However, the clinical magnitude of this association should be interpreted together with the absolute risk differences. In the adjusted cumulative incidence curves, the absolute risk differences between anaerobic antibiotic users and non-users were 0.13 percentage points at 5 years and 0.35 percentage points at 10 years. While these absolute differences appear modest, they carry substantial clinical and public health relevance due to two epidemiological contexts: the inherently low baseline incidence of maculopathy and the ubiquitous nature of antibiotic exposure nationwide. Consequently, extrapolating this risk increment across millions of users nationwide translates into a significant public health burden. Our consistent relative associations and duration-response pattern suggest that cumulative anaerobic antibiotic exposure may represent a measurable population-level risk factor for maculopathy.

To complement the cohort analysis, we conducted a nested case-control study to evaluate recent exposure to anaerobic antibiotics before maculopathy onset. Using risk-set sampling, controls were selected from individuals who were at risk at each case’s index date, allowing efficient assessment of exposure history within the underlying cohort (Pottegård, 2022). Consistent duration-response patterns were observed across Models 1, 2, and 3, with higher odds of maculopathy among patients with longer cumulative exposure to anaerobic antibiotics. This pattern remained evident after adjustment for all covariates, including major infectious diagnoses. Furthermore, formal trend analysis supported a gradual increase in risk with longer exposure duration, strengthening the evidence for a duration-response relationship in the nested case-control analysis.

As a sensitivity analysis, we restricted the population to individuals aged ≥40 years, because maculopathy is more commonly observed in middle-aged and older adults and the incidence increases with age (Ma et al., 2024). Although this restriction changed the size and composition of the study population, the results remained generally consistent with the primary analysis. This finding supports the robustness of the observed association in an age group with greater clinical relevance for maculopathy. Several additional sensitivity analyses also supported the robustness of the findings. In the cohort analysis, Fine-Gray competing risk models were applied to account for death as a competing event. Although the magnitude of the association was generally attenuated compared with the main Cox models - as is typical when accounting for competing mortality risks-a positive trend across cumulative exposure duration remained evident. In the nested case-control analysis, extending the exposure assessment window from 1 year to 2 and 3 years resulted in stronger OR estimates, supporting the consistency of the association over longer exposure windows. When lag times of 30, 90, and 180 days were applied, some estimates lost statistical significance, likely reflecting reduced sample size and fewer eligible exposure events after applying lag periods. Nevertheless, the estimates remained in the same direction, and formal trend analyses continued to show statistically significant duration-response relationships. Overall, these sensitivity analyses suggest that the observed association was not dependent on a single analytic specification.

To further examine whether the observed association was specific to antibiotics with anaerobic activity, we conducted an negative control comparator analysis using first-generation cephalosporins, which have relatively limited anaerobic activity. First-generation cephalosporin exposure was not significantly associated with incident maculopathy in either the overall exposure analysis or the duration-stratified analysis. These findings suggest that the association observed in the primary analysis was less likely to reflect antibiotic exposure in general and was more apparent for antibiotics with anaerobic activity. In addition, we conducted exploratory analyses of individual anaerobic antibiotics. Although estimates for most individual agents were limited by small numbers of exposed cases, amoxicillin/clavulanate and metronidazole had sufficient exposure frequency for meaningful estimation and showed results consistent with the primary findings: for amoxicillin/clavulanate, HR of 1.07 (95% CI, 1.02–1.11) and OR of 1.04 (95% CI, 1.02–1.07); for metronidazole, HR of 1.11 (95% CI, 1.07–1.16) and OR of 1.05 (95% CI, 1.01–1.09). The finding for metronidazole is particularly relevant because of its relatively selective anti-anaerobic activity. Together with the null findings for first-generation cephalosporins as negative control, these results provide supportive evidence that the observed association may be more closely related to anti-anaerobic activity than to antibiotic exposure in general.

This study has several limitations. First, the NHIS-NSC claims data lacked information on several potential confounders, including smoking, BMI, sun exposure, and genetic risk. Although residual confounding cannot be excluded, E-value analyses provided quantitative context for this concern. An E-value of 1.79 for the longest cumulative exposure category suggests that an unmeasured confounder would need a relatively strong association with both exposure and outcome to nullify our findings, making it less likely that residual confounding alone accounts for the observed risk. Second, this claims-based observational study was not designed to directly evaluate biological mechanisms. Therefore, the mechanisms underlying the observed association remain unclear and require further experimental investigation. Third, exposure classification has limitations. Antibiotics classified as having anti-anaerobic activity may also affect aerobic bacteria, and potential heterogeneity by individual agents or dose levels could not be fully assessed. In addition, cumulative exposure duration was based on dispensed prescription days, which may not fully capture detailed prescription patterns or actual medication adherence. In the cohort analysis, exposure was defined during the baseline assessment period and was not updated during follow-up. Therefore, the findings should be interpreted as reflecting the overall association between anaerobic antibiotic exposure and maculopathy. Finally, the matched cohort had a relatively young age distribution, which may limit generalizability to older adults at higher risk for maculopathy, although sensitivity analysis restricted to individuals aged ≥40 years showed generally consistent results.

Several strengths characterize this study. First, we used large-scale nationally representative claims data, which allowed us to reflect real-world clinical practice and examine the association between anaerobic antibiotic use and maculopathy at the population level. The longitudinal nature of the data also enabled assessment of cumulative antibiotic exposure and subsequent long-term outcomes. Second, our study moved beyond conventional broad- or narrow-spectrum classifications and chemical structure-based approaches by focusing on antibiotics with activity against anaerobes. This spectrum-oriented approach may provide additional insight into the potential role of anaerobe-targeting antibiotic exposure in maculopathy. Third, we employed two complementary study designs: a retrospective cohort design to assess long-term cumulative exposure and a nested case–control design to evaluate recent exposure before maculopathy onset. Together, these approaches provided complementary evidence for the observed association across different exposure windows and analytic perspectives.

## 5 Conclusion

In this nationwide study, we evaluated whether anaerobic antibiotic exposure influences the risk of maculopathy through alterations in the gut microbiome, focusing on antibiotics with activity against anaerobes. The use of complementary study designs—a retrospective cohort study and a nested case-control study—strengthened the robustness of the findings that exposure to anaerobic antibiotics was associated with a statistically significant, duration-dependent increase in the risk of maculopathy. These results suggest the biological plausibility of microbiome-mediated retinal vulnerability within the emerging gut-eye axis framework, although further experimental and clinical studies are warranted to fully elucidate the underlying causal mechanisms.

## Data Availability

The datasets generated and/or analyzed during the current study are not publicly available due to data protection regulations and the data access policy of NHIS, but are available from the corresponding author on reasonable request. Requests to access the datasets should be directed to jechung@hanyang.ac.kr.
