# Supplementary material for: Anaerobic antibiotic exposure and risk of maculopathy: a nationwide dual design study
Source: Front Pharmacol. 2026 Aug 21;17:1840229. doi: 10.3389/fphar.2026.1840229 (PMC13541669; doi:10.3389/fphar.2026.1840229)
Supplement: Supplementary file 1 [file Table1.docx]

Supplementary Material

**Supplementary Table S1.** Definition of ocular procedures

| Procedures | Code |
| --- | --- |
|  |  |
| Vitreous Aspiration | S5050 |
| Surgery for Glaucoma | S5041, S5042, S5043, S5033, S5047, S5049, S5048, S5040, S5039, S5038 |
| Suture Repairing of Iris, Ciliary Body | S5035 |
| Synechiolysis | S5021 |
| Resection of Vitreous Band Under Microscopy | S5060 |
| Anterior Chamber Irrigation | S5091 |
| Anterior Chamber Paracentesis | S5080 |
| Intravitreal Injection | S5070 |
| Resection of Vitreous Band | S5071, S5072 |
| Surgery for Cataract or Lens | S5111, S5119, S5112, S5110 |
| Intraocular Lens Implantation or Exchange | S5117, S5116, S5118 |
| Vitrectomy | S5121, S5122 |
| Retinal Detachment Surgery | S5130 |
| Cryopexy for Retinal Tear | S5140 |
| Periretinal Membrane Peeling | S5145 |
| Dissection of Optic Nerve Sheath | S5150 |

**Supplementary Table S2.** Definition of retinal diseases having macular complications

| Diagnosis | Code (ICD-10) |
| --- | --- |
|  |  |
| Other retinal vascular occlusions | H34.8 |
| Central retinal vein occlusion | H34.81 |
| Venous engorgement | H34.82 |
| Tributary (branch) retinal vein occlusion | H34.83 |
| Unspecified retinal vascular occlusion | H34.9 |
| Diabetic retinopathy | H36.0 |

**Supplementary Table S3.** Definition of comorbidities

| Diagnosis | | Code (ICD-10) |
| --- | --- | --- |
|  |  |  |
| Diabetes mellitus | Type 1 diabetes mellitus | E10 |
|  | Type 2 diabetes mellitus | E11 |
|  | Malnutrition-related diabetes mellitus | E12 |
|  | Other specified diabetes mellitus | E13 |
|  | Unspecified diabetes mellitus | E14 |
| Cataract | Senile cataract | H25 |
|  | Other cataract | H26 |
|  | Diabetic cataract | H28.0 |
|  | Cataract in other endocrine, nutritional and metabolic diseases | H28.1 |
|  | Cataract in other diseases classified elsewhere | H28.2 |
| Glaucoma | Glaucoma | H40 |
|  | Glaucoma in diseases classified elsewhere | H42 |
| Hypertension | Essential (primary) hypertension | I10 |
|  | Hypertensive heart disease | I11 |
|  | Hypertensive renal disease | I12 |
|  | Hypertensive heart and renal disease | I13 |
|  | Secondary hypertension | I15 |
| Dyslipidemia | Disorders of lipoprotein metabolism and other lipidaemias | E78 |
| Chronic kidney disease | Chronic kidney disease | N18 |

**Supplementary Table S4.** List of oral or Ophthalmic corticosteroid

| Classification | Drugs |
| --- | --- |
| Oral corticosteroid | Beclomethasone |
|  | Betamethasone |
|  | Budesonide |
|  | Deflazacort |
|  | Dexamethasone |
|  | Fludrocortisone |
|  | Hydrocortisone |
|  | Methylprednisolone |
|  | Prednisolone |
|  | Prednisone |
|  | Triamcinolone |
| Ophthalmic corticosteroid | Budesonide |
|  | Betamethasone |
|  | Dexamethasone |
|  | Fluorometholone |
|  | Loteprednol |
|  | Prednisolone |
|  | Rimexolone |
|  | Triamcinolone |

**Supplementary Table S5.** Risk of maculopathy associated with anaerobic antibiotics in the matched cohort over 40 years old.

| Group | Group size (n) | Events (n) | Follow-up time (person-years) | HR (Model 1)^a^  (95% CI) | HR (Model 2)^b^  (95% CI) | HR (Model 3)^c^  (95% CI) | ARDs^d^ (%) | |
| --- | --- | --- | --- | --- | --- | --- | --- | --- |
|  |  |  |  |  |  |  | at 5 years | at 10 years |
| Non-users | 156,921 | 26,958 | 1,822,489.2 | Ref | Ref | Ref | Ref | Ref |
| Users | 156,921 | 30,948 | 1,811,190.2 | 1.16 (1.14–1.18) | 1.16 (1.10–1.21) | 1.09 (1.04–1.15) | 0.20 | 0.53 |
| 1–14 days | 123,099 | 23,700 | 1,428,055.3 | 1.14 (1.12–1.17) | 1.15 (1.10–1.21) | 1.09 (1.04–1.15) | 0.18 | 0.47 |
| 15–28 days | 23,975 | 4,988 | 273,790.0 | 1.18 (1.13–1.23) | 1.16 (1.08–1.24) | 1.08 (1.01–1.16) | 0.21 | 0.54 |
| 29–56 days | 8,044 | 1,853 | 90,036.1 | 1.33 (1.23–1.43) | 1.32 (1.20–1.45) | 1.21 (1.10–1.33) | 0.42 | 1.12 |
| ≥57 days | 1,803 | 407 | 19,308.8 | 1.38 (1.17–1.62) | 1.36 (1.14–1.63) | 1.25 (1.04–1.50) | 0.59 | 1.56 |
| Trend analysis (per category increase) | | | | 1.08 (1.07–1.10) | 1.06 (1.05–1.08) | 1.03 (1.02–1.04) | - | - |

ARD, absolute risk difference; CI, confidence interval; HR, hazard ratio.

^a^Model 1 was the crude model without covariate adjustment.

^b^Model 2 was adjusted for age, sex, residential area, economic status, ophthalmology visits, hospitalization, diabetes mellitus, glaucoma, cataract, hypertension, dyslipidemia, chronic kidney disease, and use of oral or ophthalmic corticosteroids.

^c^Model 3 was adjusted for all variables included in Model 1 plus major infectious diagnoses, including respiratory tract infections, intra-abdominal infections, urinary tract infections, intestinal infections, and skin, soft tissue, bone, and joint infections.

^d^Absolute risk differences at 5 and 10 years were derived from the adjusted cumulative incidence curves estimated using Cox model 3.

**Supplementary Table S6.** Fine-Gray competing risk analysis of the association between cumulative anaerobic antibiotic exposure and incident maculopathy.

| Group | Group size  (n) | Maculopathy  events (n) | Death  events (n) | Follow-up time (person-years) | sHR (Model 1)^a^  (95% CI) | sHR (Model 2)^b^  (95% CI) | sHR (Model 3)^c^  (95% CI) |
| --- | --- | --- | --- | --- | --- | --- | --- |
| Non-users | 326,436 | 34,674 | 20,335 | 4,010,572.3 | Ref | Ref | Ref |
| Users | 326,436 | 41,115 | 20,377 | 3,981,421.2 | 1.12  (1.19–1.21) | 1.15  (1.12–1.18) | 1.04  (1.04–1.10) |
| 1–14 days | 245,020 | 30,923 | 15,396 | 2,990,143.3 | 1.19  (1.18–1.20) | 1.14  (1.11–1.18) | 1.07  (1.04–1.10) |
| 15–28 days | 51,206 | 6,790 | 3,352 | 621,191.7 | 1.26  (1.23–1.29) | 1.18  (1.14–1.23) | 1.09  (1.04–1.13) |
| 29–56 days | 21,501 | 2,681 | 1,240 | 261,913.8 | 1.24  (1.19–1.29) | 1.28  (1.21–1.35) | 1.16  (1.09–1.23) |
| ≥ 57 days | 8,709 | 721 | 389 | 108,172.4 | 0.95  (0.88–1.02) | 1.30  (1.18–1.44) | 1.16  (1.05–1.28) |
| Trend analysis (per category increase) | | | | | 1.11  (1.10–1.12) | 1.08  (1.06–1.09) | 1.04  (1.03–1.06) |

CI, confidence interval; sHR, subdistribution hazard ratio.

^a^Model 1 was the crude model without covariate adjustment.

^b^Model 2 was adjusted for age, sex, residential area, economic status, ophthalmology visits, hospitalization, diabetes mellitus, glaucoma, cataract, hypertension, dyslipidemia, chronic kidney disease, and use of oral or ophthalmic corticosteroids.

^c^Model 3 was adjusted for all variables included in Model 1 plus major infectious diagnoses, including respiratory tract infections, intra-abdominal infections, urinary tract infections, intestinal infections, and skin, soft tissue, bone, and joint infections.

**Supplementary Table S7.** Maculopathy risk according to anaerobic antibiotics use in the nested case-control study over 40 years old.

| Group | Control (n) | Case (n) | OR (Model 1)^a^  (95% CI) | OR (Model 2)^b^  (95% CI) | OR (Model 3)^c^  (95% CI) |
| --- | --- | --- | --- | --- | --- |
| Non-users | 349,389 | 34,041 | Ref | Ref | Ref |
| Users | 118,571 | 12,755 | 1.10 (1.08–1.12) | 1.10 (1.07–1.12) | 1.04 (1.02–1.06) |
| 1–7 days | 66,711 | 7,086 | 1.09 (1.06–1.12) | 1.09 (1.06–1.12) | 1.03 (1.01–1.06) |
| 8–28 days | 47,088 | 5,130 | 1.11 (1.08–1.15) | 1.11 (1.07–1.14) | 1.04 (1.01–1.08) |
| ≥29 days | 4,772 | 539 | 1.17 (1.07–1.28) | 1.16 (1.06–1.27) | 1.08 (0.99–1.18) |
| Trend analysis (per category increase) | | | 1.06 (1.05–1.07) | 1.06 (1.04–1.07) | 1.02 (1.01–1.04) |

CI, confidence interval; OR, odds ratio.

^a^Model 1 was the crude model without covariate adjustment.

^b^Model 2 was adjusted for age, sex, residential area, economic status, ophthalmology visits, hospitalization, diabetes mellitus, glaucoma, cataract, hypertension, dyslipidemia, chronic kidney disease, and use of oral or ophthalmic corticosteroids.

^c^Model 3 was adjusted for all variables included in Model 1 plus major infectious diagnoses, including respiratory tract infections, intra-abdominal infections, urinary tract infections, intestinal infections, and skin, soft tissue, bone, and joint infections.

**Supplementary Table S8.** Sensitivity analyses according to extended exposure assessment windows in the nested case-control study.

| Exposure assessment | Group | Control (n) | Case (n) | OR (Model 1)^a^  (95% CI) | OR (Model 2)^b^  (95% CI) | OR (Model 3)^c^  (95% CI) |
| --- | --- | --- | --- | --- | --- | --- |
| 2 years | Non-users | 319,943 | 30,902 | Ref | Ref | Ref |
|  | Users | 237,817 | 24,874 | 1.14 (1.11–1.16) | 1.11 (1.09–1.13) | 1.06 (1.03–1.08) |
|  | 1–7 days | 107,321 | 10,887 | 1.08 (1.05–1.10) | 1.06 (1.04–1.09) | 1.02 (1.00–1.05) |
|  | 8–28 days | 104,973 | 11,282 | 1.18 (1.15–1.21) | 1.15 (1.12–1.18) | 1.09 (1.06–1.11) |
|  | ≥29 days | 25,523 | 2,705 | 1.27 (1.20–1.33) | 1.20 (1.14–1.26) | 1.11 (1.06–1.17) |
|  | Trend analysis (per category increase) | | | 1.08 (1.07–1.10) | 1.07 (1.06–1.08) | 1.04 (1.03–1.05) |
| 3 years | Non-users | 266,194 | 25,581 | Ref | Ref | Ref |
|  | Users | 291,566 | 30,195 | 1.13 (1.11–1.15) | 1.11 (1.08–1.13) | 1.05 (1.03–1.07) |
|  | 1–7 days | 116,083 | 11,625 | 1.06 (1.04–1.09) | 1.05 (1.03–1.08) | 1.02 (0.99–1.04) |
|  | 8–28 days | 133,346 | 14,059 | 1.16 (1.13–1.19) | 1.13 (1.11–1.16) | 1.07 (1.04–1.10) |
|  | ≥29 days | 42,137 | 4,511 | 1.27 (1.22–1.32) | 1.22 (1.17–1.27) | 1.13 (1.08–1.17) |
|  | Trend analysis (per category increase) | | | 1.08 (1.07–1.09) | 1.07 (1.05–1.08) | 1.04 (1.03–1.05) |

CI, confidence interval; OR, odds ratio.

^a^Model 1 was the crude model without covariate adjustment.

^b^Model 2 was adjusted for age, sex, residential area, economic status, ophthalmology visits, hospitalization, diabetes mellitus, glaucoma, cataract, hypertension, dyslipidemia, chronic kidney disease, and use of oral or ophthalmic corticosteroids.

^c^Model 3 was adjusted for all variables included in Model 1 plus major infectious diagnoses, including respiratory tract infections, intra-abdominal infections, urinary tract infections, intestinal infections, and skin, soft tissue, bone, and joint infections.

**Supplementary Table S9.** Sensitivity analyses applying lag times in the nested case-control study.

| Lag time | Group | Control (n) | Case (n) | OR (Model 1)^a^  (95% CI) | OR (Model 2)^b^  (95% CI) | OR (Model 3)^c^  (95% CI) |
| --- | --- | --- | --- | --- | --- | --- |
| 30 days | Non-users | 407,505 | 39,960 | Ref | Ref | Ref |
|  | Users | 150,255 | 15,816 | 1.13 (1.10–1.15) | 1.10 (1.08–1.12) | 1.04 (1.02–1.06) |
|  | 1–7 days | 81,622 | 8,533 | 1.10 (1.07–1.13) | 1.08 (1.05–1.11) | 1.03 (1.00–1.06) |
|  | 8–28 days | 59,703 | 6,363 | 1.16 (1.13–1.20) | 1.12 (1.09–1.16) | 1.06 (1.02–1.09) |
|  | ≥29 days | 8,930 | 920 | 1.26 (1.16–1.36) | 1.17 (1.08–1.27) | 1.09 (1.01–1.18) |
|  | Trend analysis (per category increase) | | | 1.08 (1.07–1.10) | 1.06 (1.05–1.08) | 1.03 (1.01–1.04) |
| 90 days | Non-users | 426,927 | 41,894 | Ref | Ref | Ref |
|  | Users | 130,833 | 13,882 | 1.14 (1.12–1.17) | 1.11 (1.09–1.14) | 1.05 (1.03–1.08) |
|  | 1–7 days | 74,027 | 7,837 | 1.12 (1.09–1.15) | 1.10 (1.07–1.13) | 1.05 (1.02–1.08) |
|  | 8–28 days | 50,100 | 5,364 | 1.17 (1.13–1.21) | 1.13 (1.09–1.17) | 1.06 (1.03–1.10) |
|  | ≥29 days | 6,706 | 681 | 1.26 (1.15–1.38) | 1.17 (1.06–1.29) | 1.09 (0.99–1.20) |
|  | Trend analysis (per category increase) | | | 1.09 (1.07–1.10) | 1.07 (1.05–1.08) | 1.03 (1.02–1.05) |
| 180 days | Non-users | 458,890 | 45,373 | Ref | Ref | Ref |
|  | Users | 98,870 | 10,403 | 1.12 (1.09–1.15) | 1.09 (1.06–1.12) | 1.04 (1.01–1.06) |
|  | 1–7 days | 60,047 | 6,276 | 1.10 (1.06–1.13) | 1.08 (1.04–1.11) | 1.03 (0.99–1.06) |
|  | 8–28 days | 35,153 | 3,766 | 1.16 (1.11–1.21) | 1.12 (1.07–1.16) | 1.06 (1.01–1.10) |
|  | ≥29 days | 3,670 | 361 | 1.25 (1.10–1.41) | 1.16 (1.02–1.32) | 1.09 (0.96–1.24) |
|  | Trend analysis (per category increase) | | | 1.08 (1.06–1.10) | 1.06 (1.04–1.08) | 1.03 (1.01–1.05) |

CI, confidence interval; OR, odds ratio.

^a^Model 1 was the crude model without covariate adjustment.

^b^Model 2 was adjusted for age, sex, residential area, economic status, ophthalmology visits, hospitalization, diabetes mellitus, glaucoma, cataract, hypertension, dyslipidemia, chronic kidney disease, and use of oral or ophthalmic corticosteroids.

^c^Model 3 was adjusted for all variables included in Model 1 plus major infectious diagnoses, including respiratory tract infections, intra-abdominal infections, urinary tract infections, intestinal infections, and skin, soft tissue, bone, and joint infections.
